# New Mechanisms to Explain the Effects of Added Lactose Fines on the Dispersion Performance of Adhesive Mixtures for Inhalation

**DOI:** 10.1371/journal.pone.0087825

**Published:** 2014-01-28

**Authors:** Floris Grasmeijer, Anne J. Lexmond, Maarten van den Noort, Paul Hagedoorn, Anthony J. Hickey, Henderik W. Frijlink, Anne H. de Boer

**Affiliations:** 1 Department of Pharmaceutical Technology and Biopharmacy, University of Groningen, Groningen, The Netherlands; 2 Center for Aerosol and Nanomaterials Engineering, RTI International, Research Triangle Park, North Carolina, United States of America; Massey University, New Zealand

## Abstract

Fine excipient particles or ‘fines’ have been shown to improve the dispersion performance of carrier-based formulations for dry powder inhalation. Mechanistic formulation studies have focussed mainly on explaining this positive effect. Previous studies have shown that higher drug contents may cause a decrease in dispersion performance, and there is no reason why this should not be true for fines with a similar shape, size and cohesiveness as drug particles. Therefore, the effects on drug detachment of ‘fine lactose fines’ (FLF, X_50_ = 1.95 µm) with a similar size and shape as micronised budesonide were studied and compared to those of ‘coarse lactose fines’ (CLF, X_50_ = 3.94 µm). Furthermore, interactions with the inhalation flow rate, the drug content and the mixing order were taken into account. The observed effects of FLF are comparable to drug content effects in that the detached drug fraction was decreased at low drug content and low flow rates but increased at higher flow rates. At high drug content the effects of added FLF were negligible. In contrast, CLF resulted in higher detached drug fractions at all flow rates and drug contents. The results from this study suggest that the effects of fines may be explained by two new mechanisms in addition to those previously proposed. Firstly, fines below a certain size may increase the effectiveness of press-on forces or cause the formation of strongly coherent fine particle networks on the carrier surface containing the drug particles. Secondly, when coarse enough, fines may prevent the formation of, or disrupt such fine particle networks, possibly through a lowering of their tensile strength. It is recommended that future mechanistic studies are based on the recognition that added fines may have any effect on dispersion performance, which is determined by the formulation and dispersion conditions.

## Introduction

The dispersion performance of adhesive mixtures for inhalation may be modified by the addition of a third, fine particulate component or ‘fines’ [1]. The resulting powders are often referred to as tertiary blends. Few techniques in the preparation of adhesive mixtures for inhalation have been given as much attention in scientific literature as the addition of fines, and an excellent overview of the majority of this literature is given by Jones and Price [2]. Fines generally improve the dispersion performance of adhesive mixtures, and different hypotheses have been proposed to explain this finding (Table 1).

**Table 1:** Hypotheses concerning the working mechanism of added lactose fines.

Hypothesis	Explanation	Reference
Active sites hypothesis	Fines occupy so-called ‘active sites’ on the carrier surface, leaving only weaker binding sites available for the drug particles to bind to.	[7]
Agglomeration hypothesis	Fines form agglomerates, multiplets or multi-layers with drug particles, which are supposedly more easily detached from the carrier surface.	[7], [8]
Buffer hypothesis	Fines coarser than the drug particles may act as a buffer between colliding carrier particles and protect drug particles from press-on forces during mixing.	[11]
Fluidisation hypothesis	Fines increase the tensile strength of the bulk powder, which increases the minimum energy required for fluidisation and thus the energy available for dispersion.	[20]

Obtaining solid experimental support for the proposed hypotheses has proven to be a true challenge. As a result, the exact working mechanisms of added fines are largely unknown to date. This is not only the result of a limited number of techniques available to measure relevant powder properties. Interactions between formulation and dispersion variables also greatly add to the challenge [3]. This was, for example, shown in a study by Jones et al., in which the effect of lactose fines on dispersion performance was studied in relation to the drug content, mixing time and mixing order of the drug and fines [4]. The influence of any of these variables was apparently dependent on the levels of the other variables taken into account. Thus, interactions may explain why contradictory results have been obtained between studies on the working mechanisms of fines that were performed under different conditions, and hence, why the plethora of available data has not led to a fundamental understanding of powder performance [2].

The addition of lactose fines to adhesive mixtures in essence does not differ from an increase in drug content, since both result in a higher total amount of fines. Especially when the lactose fines have roughly the same size distribution, shape and cohesiveness as the drug particles, an effect of added fines on dispersion performance similar to that of drug content may be expected, despite a difference in chemical composition [5]. This means that also the same mechanisms might play a role.

In a previous study we have shown that the effects of drug content on dispersion performance may be explained by multiple mechanisms and that interactions likely result from a shift in their balance [6]. Two of the mechanisms used to explain drug content effects have also been proposed as working mechanisms for the effect of fines in the agglomeration and active sites hypothesis (Table 1). However, under certain conditions increasing the drug content resulted in a lower dispersion performance. Such a negative effect cannot be explained by agglomeration or the saturation of active sites alone, since these mechanisms would lead to a better dispersion performance [7], [8]. It was proposed that the negative effect of drug content on dispersion performance was caused by an increased effectiveness of press-on forces during mixing, since drug particles filled up carrier surface irregularities at higher contents and, thus, exhibited greater susceptibility to compressive mixing forces. Furthermore, the formation of strongly coherent drug particle networks on the carrier surface was observed, from which the drug may be difficult to detach. The inhalation flow rate interacts with drug content in both quantitative and qualitative ways and is, therefore, likely to cause a shift in the balance between the mechanisms in play.

Studies described in this paper address the hypotheses that, analogous to the effect of drug content, the effect of added lactose fines on the dispersion performance of adhesive mixtures can be explained by a balance between multiple mechanisms and that these mechanisms may include an increase of the effectiveness of press-on forces or the formation of coherent networks. For this purpose, the effects of added lactose fines with a median particle size close to that of the drug (i.e. <2 µm) on the dispersion performance of adhesive mixtures with different budesonide contents were studied over a range of flow rates. Furthermore, interactions with the size distribution of the fines and mixing order of the drug and fines were studied. These variables are expected to change especially the contribution of the ‘buffer mechanism’ and the ‘active sites mechanism’, respectively, to the overall effect of added fines.

## Materials and Methods

### Starting materials

Alpha-lactose monohydrate of different grades was obtained from DFE Pharma (Goch, Germany). Pharmatose 80 M was used to obtain a coarse size fraction of carrier particles, whereas Respitose ML006 was micronised to obtain lactose with a size distribution roughly comparable to that of the drug (i.e. fine lactose fines, FLF). Lactose fines coarser than the drug (i.e. coarse lactose fines, CLF) were obtained by micronisation of a Lactohale product (Borculo Domo Ingradients, Borculo, The Netherlands). The FLF and CLF had been stored for at least one year under environmental conditions before use. During this period they have been exposed multiple times to air with a relative humidity varying closely around 50%, and therefore, any amorphous surfaces formed during the milling process had likely recrystallised over time. Micronised budesonide (Fagron, The Netherlands) was the drug used in this study. To break up larger agglomerates, the drug and lactose fines were passed through a 90 µm test sieve at least several days prior to preparation of the mixtures.

### Carrier classification

Pharmatose 80 M was sieved for 20 minutes with a vibratory sieve (Retsch AS 200 control, Germany) to obtain a carrier size fraction of 250–315 µm. The vibratory sieving procedure was then followed by 15 minutes of air jet sieving (Alpine A200, Augsburg, Germany) to remove as many intrinsic lactose fines from the carrier material as possible. The carrier classification procedure is the same as that used in the study on drug content effects [6], which allows the data from both studies to be compared.

### Laser diffraction analysis

Particle size distributions of the mixture components were measured with the HELOS BF laser diffractometer after dispersion with a RODOS powder disperser at 3 bar (Sympatec, Clausthal-Zellerfeld, Germany). For the drug and lactose fines a 100 mm lens with a measuring range of 0.5/0.9–175 µm was used, whereas the carrier material was measured with a 500 mm lens (4.5–875 µm measuring range). Data are based on the Fraunhofer theory. Increasing the dispersion pressure to 5 bar did not result in a change of the measured particle size distributions, which indicates that the size distributions of the approximate primary particles were obtained at 3 bar. Results are the mean of three measurements.

### Blend preparation

Blends were prepared at ambient conditions in batches of 25 g. Different amounts of the drug and lactose fines were ‘sandwiched’ simultaneously between two equal parts of the carrier material in a 160 cc stainless steel mixing vessel and gently pre-mixed with a spatula for several orbits. Mixing was then continued with a Turbula blender (WA Bachhofen, Basel, Switzerland) operated at 90 rpm for 5 or 10 minutes. Mixing order experiments involved blending of the carrier material with either budesonide or lactose fines for 5 minutes. Subsequently, the other fine particulate component (lactose fines or budesonide, respectively) was added and mixing was continued for another 5 minutes.

### Content uniformity testing

Blends were tested for content uniformity by taking 20 samples of 25 mg from randomly chosen positions in the powder bed. The content of the samples was determined as described under ‘sample analysis’. Blends were considered homogeneous and suitable for further testing at relative standard deviations (RSDs) of the content <3%.

### Scanning electron microscopy (SEM)

Scanning electron micrographs were obtained with a JSM-6301F (Jeol, Japan) at an acceleration voltage of 2 or 3 kV. Samples were mounted on an aluminium sample holder by means of conducting double sided adhesive tape. Loose particles were tapped off very gently to leave the drug-fines adherence to the remaining carrier particles on the sample holder unchanged. The specimens were then sputter coated with 20 nm of a gold/palladium alloy (120B, Balzers AG, Liechtenstein).

### Drug detachment experiments

Drug detachment experiments were performed as described previously [6]. In summary, inhalation experiments were performed with a classifier based test inhaler [9] at fixed inhalation flow rates from 10 to 60 L/min. The resistance of the test inhaler is 0.056 kPa^0.5^ min L^−1^, and therefore, the inhalation flow rates correspond to pressure drops of 0.2–11.4 kPa. For each measurement an accurately measured amount of 25 mg of an adhesive mixture was loaded by hand into the classifier of the test inhaler. Carrier particles were collected from the inhaler's classifier after the experiment to measure the residual (non-detached) drug content (carrier residue, CR) and CR-values were corrected for minor carrier passage fractions towards 100% retention. Drug detachment was calculated as 100-CR. Results are the mean of five measurements.

The inhalation conditions may deviate from those relevant to the practice of dry powder inhalation, because the purpose is not to simply conduct functionality or effect experiments, but rather to obtain a mechanistic insight into the effects of added lactose fines. Related to this is also the choice for drug detachment as a measure of dispersion performance. Changes in drug detachment may not equal changes in the fine particle fraction (the dispersion performance characteristic most relevant to the ultimate goal of dry powder inhalation: good patient therapy), since large agglomerates may be detached too that are not further dispersed. However, drug detachment from lactose carriers is a prerequisite for obtaining a fine particle fraction, and therefore, a mechanistic understanding of how lactose fines affect this process is highly relevant. Furthermore, changes in drug detachment can directly be related to changes in the separation and adhesion force distributions throughout the mixture during inhalation [9], which may facilitate the interpretation of data and enhance the resulting mechanistic insights.

### Sample analysis

Samples were analysed by spectrophotometry to determine their drug content (Unicam UV-500, ThermoSpectronic, Cambridge, UK). Budesonide mixtures were suspended in ethanol and after 1 hour suspended lactose was removed by centrifugation at 3000 rpm for 5 minutes (Hettich Rotanta D-7200, Hettich AG, Switzerland). The resulting clear solution was then analysed at a wavelength of 243 nm.

## Results

### Laser diffraction analysis

The particle size distributions of the mixture components from laser diffraction analysis are presented in Table 2. The particle size distribution of the FLF is comparable to that of the drug, with the X_50_ being only 0.37 µm higher. The CLF are markedly coarser, with an X_50_ more than two times higher than those of the FLF and the drug. No particles <10 µm were measured in the sieved carrier material after RODOS dispersion. Based on the X_50_-values of the fine components the total carrier surface coverages of the different formulations were calculated, which are presented in Table 3.

**Table 2:** Particle size distributions of the mixture components (average (SD); n = 3).

Component	X_10_ (µm)	X_50_ (µm)	X_90_ (µm)
Budesonide	0.70 (0.00)	1.58 (0.02)	3.10 (0.03)
FLF	0.86 (0.01)	1.95 (0.00)	3.48 (0.01)
CLF	1.22 (0.02)	3.94 (0.07)	9.15 (0.24)
Carrier	241.6 (0.1)	344.8 (0.2)	475.8 (1.1)

**Table 3:** Formulations studied and their total carrier surface coverage (CC) by fine components.

Formulation	CC (%)*
0.4% Bud	28
4% Bud	283
0.4% Bud +4% FLF	217
0.4% Bud +4% CLF	124
4% Bud +4% FLF	471
4% Bud +4% CLF	378

* The carrier surface coverage is calculated as explained previously [21]. For these calculations, the X_50_-values of the fine components from Table 2 were used and densities of budesonide and alpha-lactose monohydrate were considered to be 1.25 [6] and 1.53 g/cm^3^, respectively. The calculated values are based only on added fine components (drug and lactose fines) and do not take into account the presence of lactose fines intrinsic to the carrier material after the sieving procedure.

### Content uniformity

RSDs of the drug contents in the mixtures ranged from 0.6–2.2%. Therefore, all mixtures were considered homogeneous and suitable for the drug detachment experiments.

### SEM imaging

**Figure 1:**
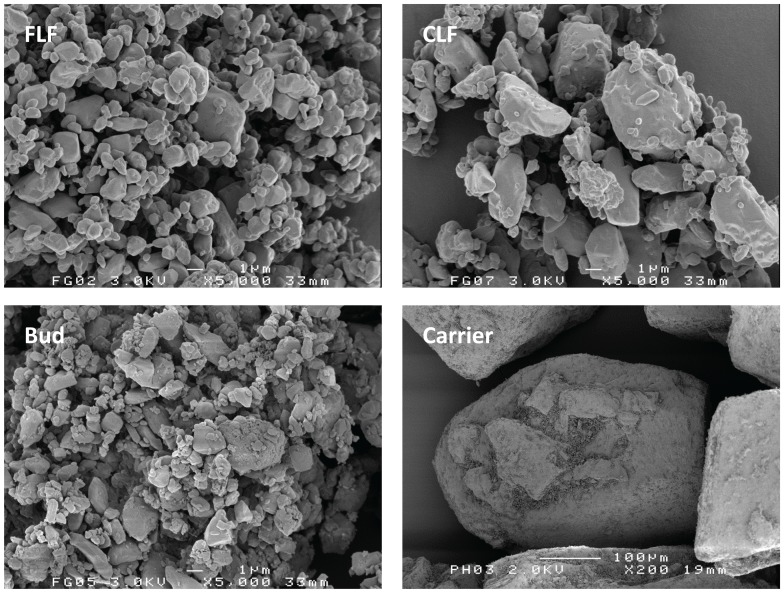
Representative SEM images of the pure mixture components.

Representative scanning electron micrographs of the pure mixture components are presented in Figure 1. Fines are visible in carrier surface irregularities of the sieved carrier particles, despite the fact that they were not measured with the laser diffraction technique. Apparently neither the double sieving procedure nor RODOS dispersion does effectively remove all of the fines from the carrier surface. The images confirm that the FLF have a size distribution similar to budesonide and that CLF are markedly coarser. The shapes of the budesonide particles roughly resemble those of the lactose fines particles.

**Figure 2:**
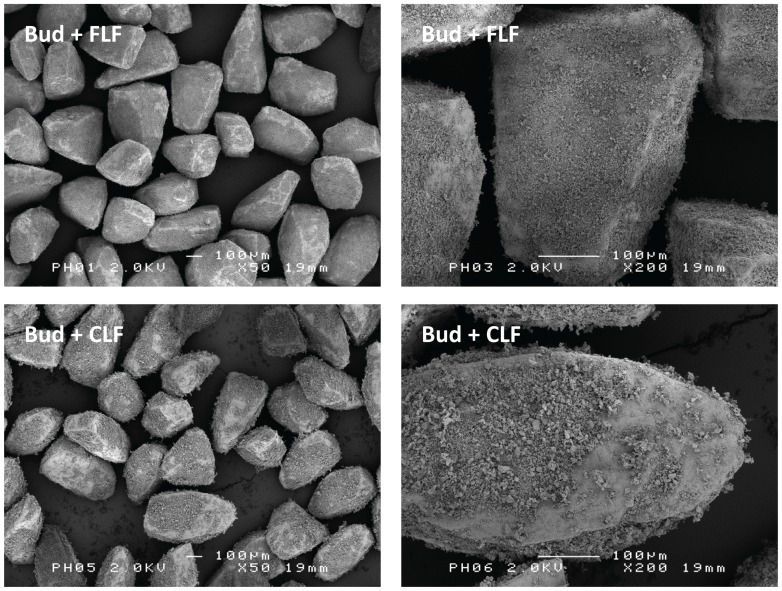
Representative SEM images of mixtures. Top: mixtures containing 4% budesonide and 4% fine lactose fines. Bottom: mixtures containing 4% budesonide and 4% coarse lactose fines. Images on the right hand side are a magnification of images on the left hand side.

SEM images of mixtures containing 4% budesonide and 4% of FLF or CLF (mixed simultaneously for 10 minutes) are presented in Figure 2. A more dense and continuous layer around the coarse carrier particles appears to be formed by FLF and budesonide than by CLF and budesonide. The typical difference in structure of the fine components between FLF and CLF has been observed with SEM for batches of 0.4 and 4% budesonide mixtures as well as for mixtures containing other drugs (salbutamol sulphate and salmeterol xinafoate; unpublished data). The images shown in Figure 2 are therefore likely to be obtained from representative samples and they are representative for different drugs as well.

### Drug detachment experiments

**Figure 3:**
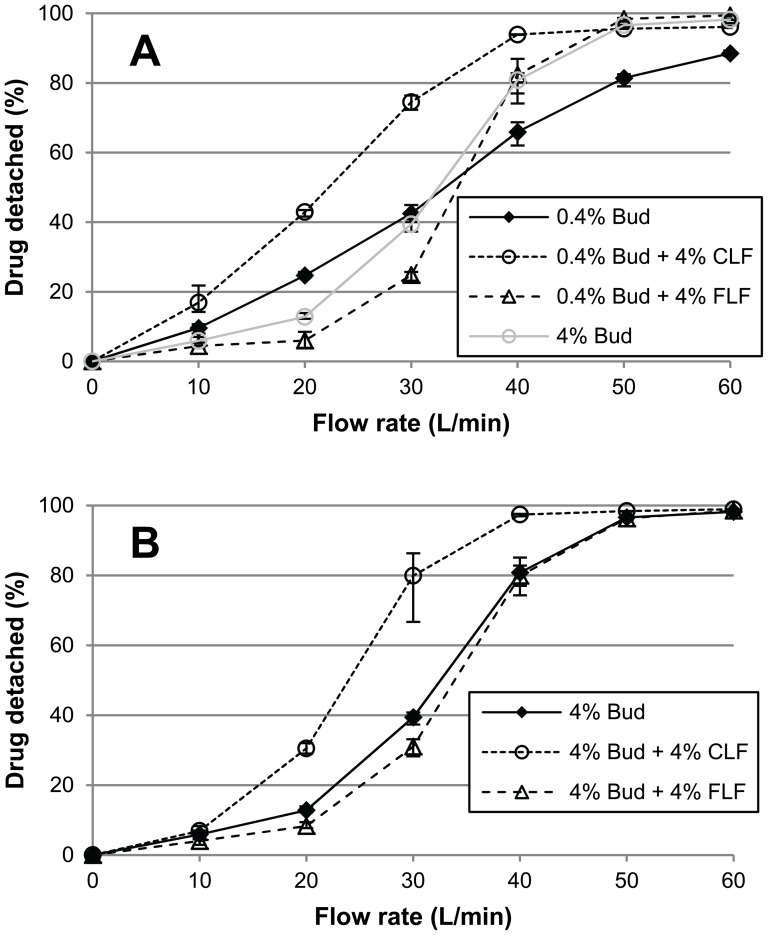
The effects of added lactose fines on drug detachment over a range of flow rates. **A**) effects of 4% fine lactose fines (FLF) or 4% coarse lactose fines (CLF) on drug detachment of 0.4% budesonide mixtures and **B**) for 4% budesonide mixtures. Error bars represent maximum and minimum values measured (n = 5).

Figure 3 shows that the effect of 4% added lactose fines on the detached drug fraction depends on the size distribution of the fines, the budesonide content and the flow rate.

#### The effect of 4% added FLF

The addition of 4% FLF to a 0.4% budesonide mixture results in a lower detached drug fraction at flow rates of 10–30 L/min (Fig. 3A). At 20 L/min this effect is most pronounced with a decrease in the detached drug fraction from 24.7% to 6%. At flow rates between 40 and 60 L/min the detached drug fraction increases compared to 0.4% budesonide alone, with a highest absolute increase of 18% occurring at 50 L/min. Hence, flow rate interacts in both qualitative and quantitative ways with the effect of added FLF on drug detachment at a low drug content. This is similar to the effects on the detached drug fraction of an increase in budesonide content from 0.4% to 4%, although then the negative effect at low flow rates is less pronounced (Fig. 3A). For 4% budesonide mixtures, the effect of adding 4% FLF is relatively small. At this drug content the detached drug fraction decreases at most 8.4% (absolute difference; Fig. 3B, 30 L/min).

#### The effect of 4% added CLF

For mixtures containing 0.4% (Fig. 3A) or 4% of budesonide (Fig. 3B), the addition of 4% CLF results in higher detached drug fractions over the range of flow rates applied. Thus, in contrast to FLF, there is no qualitative interaction between flow rate and the effect of CLF. Flow rate does interact in a quantitative way with the effect of CLF, however. The absolute increase in the detached drug fraction is highest at 30 L/min with 32% and 40.5% for budesonide contents of 0.4% and 4%, respectively. At flow rates of 10, 50 and 60 L/min the effect of CLF on the detached drug fraction is negligible for 4% budesonide mixtures.

#### The influence of the fines content

**Figure 4:**
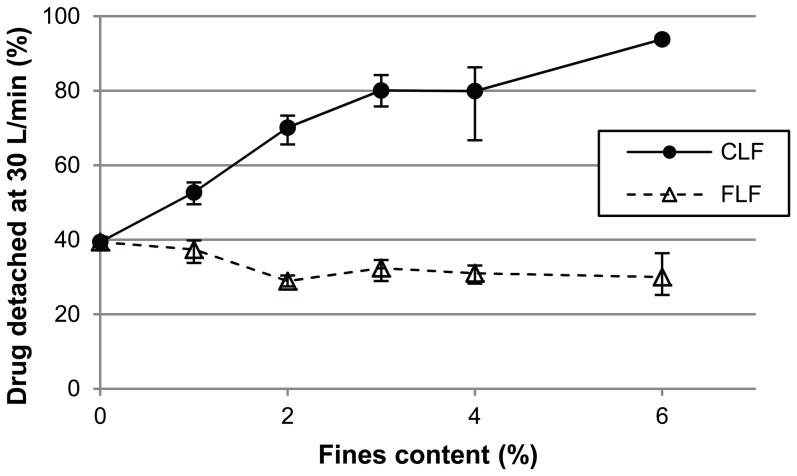
Drug detachment of 4% budesonide mixtures at 30 L/min as a function of added fines content. FLF =  fine lactose fines; CLF =  coarse lactose fines. Error bars represent maximum and minimum values measured (n = 5).

The amount of added lactose fines may influence the effect on the detached drug fraction, depending on the size distribution of the added fines. At 30 L/min, the detached drug fraction of 4% budesonide mixtures is positively and nearly linearly related to the CLF content (0–6%), whereas it is almost independent of the FLF content (Fig. 4). Some exploratory experiments revealed that the inhalation flow rate and the drug content may also be of influence. For example, at 20 L/min the detached drug fractions of 0.4% budesonide mixtures containing 0%, 0.4% or 4% fines were 24.7%, 14.4% and 6%, respectively (FLF), or 24.7%, 32.1% and 42.9%, respectively (CLF). Hence, detached drug fractions with 0.4% added fines were intermediate to those with 0% and 4% added fines at 20 L/min. However, at a flow rate of 50 L/min the effect on the detached drug fraction of 0.4% of fines was negligible (<4.2%) for both FLF and CLF, whereas the addition of 4% fines increases the detached drug fraction by 18% (see Fig. 3A).

#### The influence of mixing order on the effect of 4% added fines

**Figure 5:**
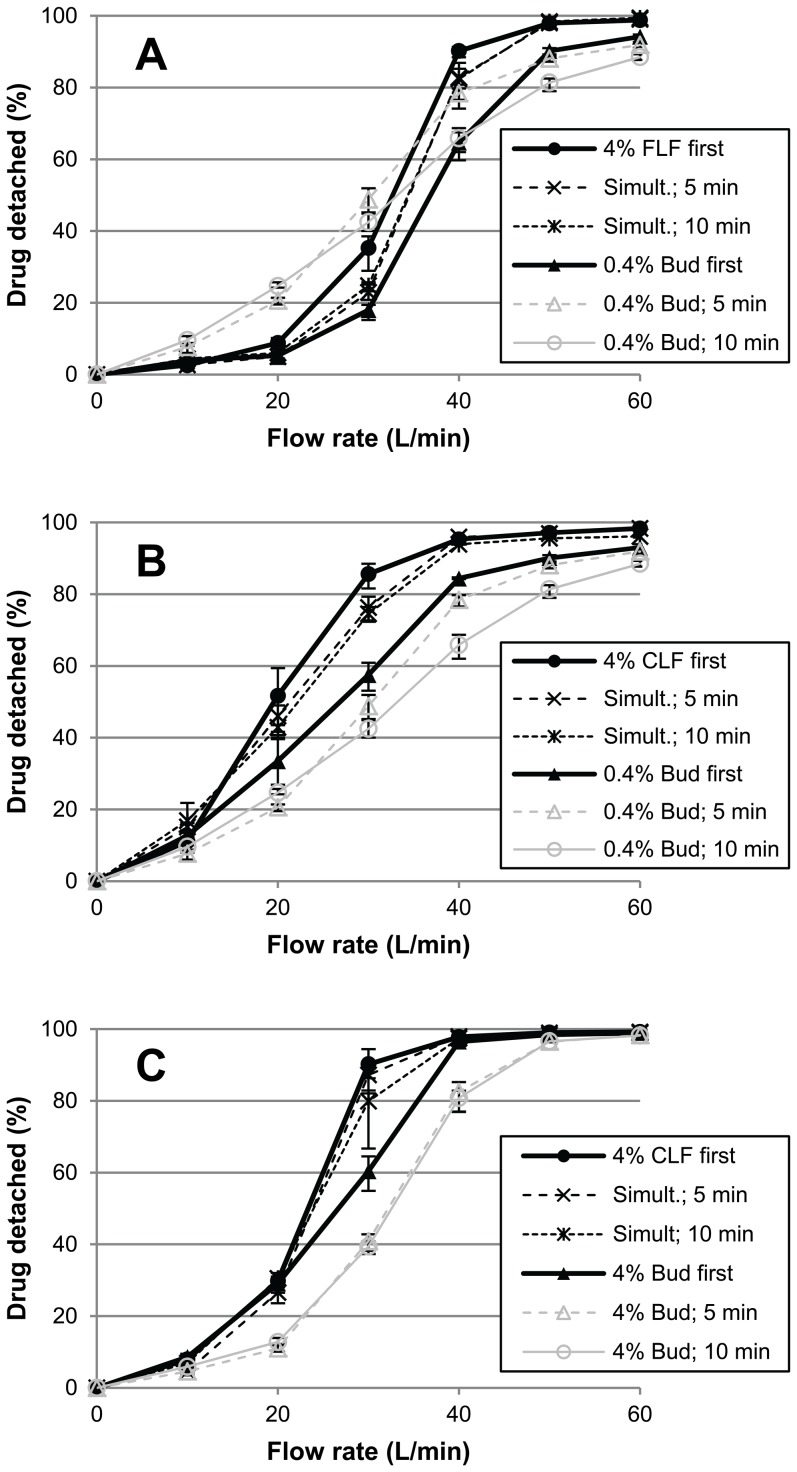
The influence of mixing order on the effects of added fines on drug detachment. **A**) mixtures containing 0.4% budesonide and 4% fine lactose fines (FLF); **B**) mixtures containing 0.4% budesonide and 4% coarse lactose fines (CLF); **C**) mixtures containing 4% budesonide and 4% CLF. Drug detachment versus flow rate profiles of mixtures containing only 0.4% or 4% of budesonide (mixed for 5 or 10 minutes) are shown as a reference. Fine components were mixed separately by mixing one component with the carrier for 5 minutes, after which the second component was added and mixing was continued for another 5 minutes. Simult.  =  drug and fines mixed simultaneously with the carrier material for 5 or 10 minutes. Error bars represent maximum and minimum values measured (n = 5).

The mixing order of the fine components interacts with the effect of 4% added fines on drug detachment from the lactose carrier. The magnitude of this interaction depends on the applied flow rate, the size distribution of the fines and the drug content (Fig. 5). In all scenarios where mixing order causes a pronounced difference in the effect of added fines on drug detachment, the largest effect on the detached drug fraction compared to the situation without added fines is obtained by mixing of the fines first. In contrast, the smallest effect on the detached drug fraction is obtained by mixing the drug first in all scenarios. At 50 and 60 L/min, the addition of 4% FLF or CLF to and subsequent 5 minutes of mixing with a 0.4% budesonide only mixture (mixed for 5 minutes) does not result in a change in the detached drug fraction (Figs. 5A and 5B, “0.4% Bud; 5 min” and “Bud first”). The absolute difference in the detached drug fraction between the fines first and budesonide first mixtures is pronounced especially at flow rates of 30 and 40 L/min for 0.4% budesonide with 4% FLF (17.3% and 25.5%, respectively, Fig. 5A) and 20 and 30 L/min for 0.4% budesonide with 4% CLF (18.2% and 28.1%, respectively, Fig. 5B). The mixing order of 4% budesonide and 4% CLF affects the detached drug fraction at a flow rate of 30 L/min (29.8% difference in the detached drug fraction, Fig. 5C), whereas at all other flow rates applied the influence of mixing order is negligible. Mixing of fines and budesonide simultaneously for 5 or 10 minutes results in detached drug fractions intermediate to those obtained by mixing the fines or budesonide first at 30 and 40 L/min (0.4% budesonide +4% FLF, Fig. 5A) or at 20 and 30 L/min (0.4% budesonide +4% CLF, Fig. 5B). At higher flow rates the simultaneous mixing of budesonide and fines results in detached drug fractions equal to those obtained by mixing the fines first for these two formulations. For the formulation containing 4% budesonide +4% CLF (Fig. 5C, 30 L/min), mixing both components simultaneously for 10 minutes results in a detached drug fraction intermediate to those obtained by mixing the fines or the drug first. Mixing both fine components simultaneously for only 5 minutes results in a detached drug fraction equal to that obtained by mixing the fines first.

## Discussion

### About the presence of intrinsic lactose fines

The presence of intrinsic lactose fines on the carrier surface after the sieving procedure (Fig. 1) has likely affected the balance of effects resulting from the addition of FLF or CLF. For example, intrinsic fines may occupy part of the active sites on the carrier surface or cause a ‘buffering effect’ during mixing of the drug with the lactose carrier. This would decrease the potential for the ‘saturation of active sites’ or a buffering effect by FLF or CLF. Therefore, it would be desirable to use more effective methods for the removal of intrinsic lactose fines. Other workers have used techniques that included submersion of the carrier material in ethanol based solutions [10], [11]. Although such techniques may result in lower intrinsic fines contents, they also change carrier surface properties like surface roughness and the degree of contamination with protein residues. This too will change to unknown extent the potential for certain effects when adding lactose fines, and therefore, such techniques are not ideal either. The carrier classification method used in this study may better represent the practical situation, since commercial carrier products will always contain a certain amount of intrinsic lactose fines too. Furthermore, the same classification method was used in a previous study on drug content effects [6], which allows a better comparison of these effects with those obtained by the addition of lactose fines in the current study.

### About the effects of FLF

The effects of FLF on the detached drug fraction are similar to drug content effects. With a drug content of 0.4%, flow rate interacts in the same qualitative way with the effects on drug detachment of 4% added FLF as with those of 3.6% added budesonide (i.e. for a total drug content of 4%, Fig. 3A). Also for three other drugs similar effects of an increase in drug content from 0.4–4% have been reported [6], even though these drugs differed in their adhesiveness and cohesiveness, particle shape and density. This suggests that these component specific properties are of minor importance to the effect of fines content on dispersion performance. It may therefore be expected that the effects of FLF obtained with budesonide in this study are representative for other drugs as well, which we have already been able to confirm for salbutamol sulphate and salmeterol xinafoate (unpublished data). The 0.4% difference in added fine particle content between FLF and budesonide is unlikely to have affected the presented finding, because minor drug content effects have been reported at drug content ranges >2% [6]. This too agrees well with the minor effects of FLF on drug detachment of 4% budesonide mixtures (Figs. 3B and 4).

The similarity between the effects of FLF and drug content on the detached drug fraction suggests that the same working mechanisms play a role. We have previously shown that drug content effects may be explained by a balance between agglomeration effects, press-on effects and the saturation of active sites [6]. Agglomeration effects and the saturation of active sites are addressed in the ‘agglomeration’ and ‘active sites’ hypothesis, respectively (Table 1). The press-on effect refers to an increase in interaction forces between the mixture constituents due to inertial and frictional forces exerted on the mixture during the blending process. A stronger effect of such press-on forces may be expected with increasing fines contents. This stronger effect is caused by fine particles filling up carrier surface irregularities, allowing the forces caused by mixing to be transferred through the powder bed and to act effectively on fine particles that would have been sheltered in the irregularities from these forces at lower fines contents. The qualitative interaction of flow rate with the effect of drug content on the detached drug fraction of 0.4% mixtures was previously explained by a change in the relevance to drug detachment of the different mechanisms that occur during mixing. At flow rates roughly <40 L/min press-on effects are most relevant or dominant, which shifts to a dominance of agglomeration effects and the saturation of active sites at higher flow rates [6]. This may also apply to the interaction of flow rate with the effect of FLF. With an initial drug content of 4%, active sites are already saturated, press-on forces are likely already maximally efficacious and the addition of FLF does not lead to pronounced agglomerate formation (Fig. 2). This may explain the minor effect of FLF on drug detachment when added to 4% budesonide mixtures.

### About the influence of the size of the added fines

The difference in effect on drug detachment between FLF and CLF is in agreement with the ‘buffer hypothesis’ as explained in Table 1. According to this hypothesis, CLF should have a more beneficial effect on the detached drug fraction than FLF, because their larger size should allow them to better protect the drug particles from press-on forces by acting as a buffer between colliding carrier particles or between their carrier particle and the wall of the mixing vessel. This would result in weaker drug-carrier interactions and thus lead to a higher chance of drug particle detachment during inhalation.

Several other explanations for the difference in effect on drug detachment between FLF and CLF may be given, however, which causes the occurrence and significance of a ‘buffer effect’ with CLF to remain unclear. Extensive, coherent networks of fine particles are formed around the coarse carrier particles at total drug and FLF contents ≥4% (Fig. 2, top and [6], Fig. 5). The formation of such particle networks is often considered as part of the ‘agglomeration hypothesis’ (Table 1). However, in contrast to single, detachable agglomerates, the detachment of particles from such networks requires ‘cracking’ or failure of some of the cohesive and adhesive bonds between the fine particles. Drug detachment thus likely depends on the tensile strength of the particle network. The tensile strength of agglomerates can be described as in Eq. 1 [12]:
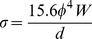
(Eq.1.)in which σ is the tensile strength of the agglomerate, φ the packing fraction (volume of particles per volume of agglomerate), W the work of adhesion or cohesion and d the particle diameter. From Eq. 1 it follows that the use of fines with a larger diameter may decrease the tensile strength of any fine particle network formed, which should benefit drug detachment. Such an effect may be expected to depend on the fines content relative to the drug content, as observed for CLF (Fig.4). This idea is supported by the work of Adi et al., who studied the effect of lactose fines with different size fractions on the dispersion performance of carrier-based and carrier-free mixtures with salmeterol xinafoate [13]. Fines with a median diameter of 7.9 µm led to a better dispersion performance of the mixtures than fines with a median diameter of 3.0 µm. For carrier-based formulations, the influence of the fines content on their effect on dispersion performance was studied. The magnitude of the effect of the coarser fines was proportional to their content over a range from 5 to 20%. These workers also observed differences in the structure of agglomerates formed and stated that an influence on the tensile strength was the most likely cause of the difference in dispersion performance between both fines size fractions. In addition to a direct particle size effect on the tensile strength of the fine powder bed, there may also be some indirect effects. Coarser fines most probably rise above carrier surface irregularities and have a higher mass and inertia. This would make them more susceptible to redistribution by frictional and inertial forces during mixing, which may prevent the formation of particle networks with a high packing fraction. Furthermore, the addition of an equal weight fraction of a coarser material represents fewer particles, which reduces the carrier coverage (Table 3) and the number of contact points that can be formed per unit agglomerate volume and thus the potential for the formation of vast and coherent particle networks. These factors may also explain the observed difference between FLF and CLF in the structure of the fine particle layer they form with the drug on the carrier surface (Fig. 2). The above hypothesis may be referred to as the ‘tensile strength hypothesis’, not to be confused with the ‘fluidisation hypothesis’ (Table 1), which refers to the tensile strength of the powder bulk. In light of the above, agglomeration (i.e. the formation of single particle-like clusters from primary particles) will be distinguished from network formation throughout the rest of this paper. Lastly, particle detachment from the lactose carrier is supposedly for the larger part caused by inertial separation forces, especially in the classifier based test inhaler used in this study [9], [14]. Inertial separation forces are proportional to d^3^, whereas most types of interaction forces are only proportional to d^1^. Hence, coarser particles will detach more easily from the carrier surface during inhalation, as was shown by Dickhof and co-workers for different budesonide size fractions [15]. It may therefore be expected that CLF detach more easily from the carrier surface than FLF. This likely increases drug detachment too as drug particles may to some extent be co-agglomerated with the fines or liberated from simultaneously ‘cracking’ particle networks. The given hypotheses to explain the difference in effect between FLF and CLF do not rely heavily on the properties of the drug component. A similar difference between the effects of CLF and FLF may therefore be expected for other drugs with different adhesion and cohesion characteristics, as has already been observed for salmeterol xinafoate (0.4% drug content only) and salbutamol sulphate (unpublished data).

### About the influence of the mixing order

The finding that the influence of the mixing order of drug and fines on the detached drug fraction depends on the flow rate, on the particle size of the fines and on the drug concentration (Fig. 5) is in agreement with results from previously presented studies. For example, Zeng et al. observed that mixing of milled lactose (5.0 µm) with the carrier material first, followed by addition of the drug resulted in a higher fine particle fraction at 60 L/min through a Rotahaler than mixing of the drug first. However, at a different flow rate (90 L/min) or when fines with a different volume median diameter (15.9 µm) were used, the influence of mixing order on the fine particle fraction was insignificant [16]. In another study Jones et al. showed that mixing order did affect the fine particle fraction of salbutamol mixtures at 60 L/min through a Rotahaler, but only at drug contents of 0.5 and 1.5% and not at drug contents of 2.5, 3.5 or 4.5% (with a carrier size fraction of 63–90 µm)[4]. These workers, furthermore, showed that the influence of mixing order was dependent on mixing time too. Because the formulation and dispersion conditions chosen in the above studies are different from those in the current study, the observed interactions with the influence of mixing order are not restricted to a specific set of formulation and dispersion conditions.

Mixing order experiments have mostly been performed to test the active sites hypothesis (Table 1) [2]. Theoretically, if fines indeed act on dispersion performance through the occupation of active sites, then mixing the fines first would maximally exploit this mechanism and should result in a more beneficial effect on dispersion performance than any other mixing order. As a result, the presence of a mixing order effect has always been interpreted as support of the active sites hypothesis, whereas the absence of such an effect has always been interpreted as a falsification of the active sites hypothesis. The occurrence of interactions with the effect of mixing order shows that the interpretation of mixing order experiments is not that straightforward, however. Several assumptions underlying the active sites hypothesis and the classical interpretation of mixing order experiments are, therefore, extensively discussed in the following sections before interpreting the results presented in Figure 5.

Firstly, once fine excipient particles bind to active sites, the new surface they form for drug particles to settle on should be less ‘active’ than the original carrier surface. If this is not the case, then binding of the fines to active sites will not result in a higher chance for drug particles to bind to sites with a lower activity. This point was addressed by Louey and Stewart, who used atomic force microscopy to measure the adhesion force between a silica sphere and the carrier surface [8]. They measured higher adhesion energies after the addition of a ternary component and reasoned that drug particles would be more likely to bind to the ternary component than to carrier sites with a low activity. As a result, it was concluded that the saturation of active sites was unlikely to be an important mechanism for the effect of added fines on dispersion performance.

Secondly, once bound to active sites, the redistribution or replacement of particles should be limited. If a highly dynamic situation exists during mixing in which fine particles are continuously redistributed to and from active sites, similar equilibrium situations may be reached independent of the mixing order of the fine components, even after mixing for a relatively short duration as in this study. The fact that drug detachment was influenced by mixing order suggests that at least a highly dynamic situation did not exist during mixing in our experiments. Especially noteworthy in this respect may be the small difference in drug detachment at 50 and 60 L/min caused by the addition of FLF or CLF to a mixture containing 0.4% budesonide mixed for 5 minutes (Figs. 5A and 5B, “0.4% Bud; 5 min” and “0.4% Bud first”). At these high flow rates it may be expected that the non-detached drug particles are attached to the most active sites. After mixing 0.4% budesonide with the carrier material for 5 minutes, continued mixing for another 5 minutes with added FLF or CLF does not change the detached drug fraction, whereas mixing the fines first does result in a 4.6–7.7% higher detached drug fraction. Because drug detachment of the ‘budesonide first’ mixture would be expected to be closer to the ‘fines first’ mixture in the case of a highly dynamic situation, at least in between the ‘fines first’ and the ‘5 minutes budesonide alone’ mixture, this may be indicative of a limited dynamic situation. Furthermore, because continued mixing of budesonide alone for a total of 10 minutes (“0.4% Bud; 10 min”) does result in a 6.7% and 3.7% lower detached drug fraction at 50 and 60 L/min, respectively, compared to only 5 minutes of mixing, the addition of fines may prevent the further distribution of drug particles towards active sites or prevent the firm compression of drug particles onto the carrier surface (i.e. onto sites previously referred to as ‘pseudo-active sites’ [17]).

Thirdly, in order for the active sites hypothesis to be relevant, active sites should have the potential to significantly influence dispersion performance. This means that: 1) if no fines are added to the mixture, a significant drug mass relative to the total drug mass should be bound to active sites; and 2) drug particles which are displaced from active sites by fines have to contribute to the dispersion performance characteristic measured (i.e. fine particle fraction or drug detachment). Regarding the first point it can be stated that with a fixed binding capacity of active sites a potentially higher drug fraction may be bound to active sites at lower drug contents. Hence, the potential contribution of the active sites mechanism to the effect of added fines on dispersion performance will be larger at lower drug contents. This may explain the influence of drug content as found by Jones et al. [4], mentioned previously, and the observation in the present study that mixing order influences the detached drug fraction at 40–60 L/min for the 0.4% budesonide mixtures (Figs. 5A and B), whereas it does not for the 4% mixture (Fig. 5C). It should be noted, however, that the binding capacity of active sites, and thus the potential for the active sites mechanism to play a significant role, depends on the definition of active sites that is used. To further explain the second point, it may be clarifying to think of the drug mass being distributed as function of the flow rate at which detachment from the carrier surface will occur. The choice of flow rate during dispersion experiments determines whether a certain shift within the mass distribution caused by the addition of fines can be measured. For example, drug particles in a binary mixture that detach at flow rates ≥60 L/min, but after the addition of lactose fines detach already at 40 L/min, do not contribute to a higher mass fraction (i.e. an improved dispersion performance) if drug detachment is only measured at flow rates ≤30 L/min. Thus, attributing a positive effect of added lactose fines on dispersion performance measured at 30 L/min to the saturation of active sites implicitly defines active sites as those sites on the carrier surface from which drug is not detached by dispersion at 30 L/min. This point also highlights the importance of the definition of active sites. In Figure 5C, the difference in the detached drug fraction at 30 L/min between the ‘fines first’ and ‘drug first’ mixing orders is the result of a shift in the drug mass distribution from the subfraction that is detached at 30–40 L/min to the subfraction detached at 20–30 L/min. Therefore, only if the definition of active sites includes that part of the carrier surface from which drug particles are not detached at 30–40 L/min this mixing order effect may be considered evidence in favour of the active sites mechanism. However, such a definition would be very broad.

Finally, the saturation of active sites should be the only mechanism of which the occurrence or significance is affected by mixing order. If this is not the case, then an observed mixing order effect does not form conclusive evidence in favour of the active sites hypothesis and the likelihood of this mechanism playing a role has to be inferred from the formulation and dispersion conditions used. Mixing order effects may reasonably be expected for at least the ‘buffer hypothesis’, the ‘tensile strength hypothesis’ and the ‘agglomeration hypothesis’ too. For example, the compression of drug particles onto the carrier surface and the formation of drug particle networks with a high tensile strength in the absence of fines (i.e. mixing the drug first) may not be fully reversible after the addition of fines. Furthermore, it may prevent the drug particles from being available for agglomeration with the added lactose fines.

It may be concluded based on the above that the mixing order data presented in Figure 5 do not provide evidence against or in favour of just one working mechanism for the effect of added fines. At best one may conclude that multiple mechanisms are likely to be relevant, which all seem to depend on the mixing order of the fine components to some extent. At low drug contents and high dispersion efficacies (i.e. flow rates), an influence of mixing order is most likely to be an indication that the active sites hypothesis could be true, but its significance relative to other mechanisms remains questionable.

### About practical implications

The results from this study show that the addition of lactose fines to adhesive mixtures for inhalation is an important technique that can serve multiple purposes. Traditionally, fines are added to carrier-based inhalation formulations solely to improve their dispersion performance. However, Figures 3 and 5 show that lactose fines may also be used to influence the flow rate dependence of the formulation's dispersion performance. This flow rate dependence of the dispersion performance should ideally be chosen such, that it compensates for the shift in drug particle deposition towards larger airways with increasing flow rate, as shown *in vivo* by Usmani et al. [18]. It then aids in obtaining a flow rate independent lung deposition or therapy. Of course, this assumes the use of a dispersion principle that effectively converts the higher kinetic energy of the air resulting from higher flow rates into higher dispersion forces. Added lactose fines, especially FLF, may furthermore be used to lower the effect on dispersion behaviour of drug content. The difference in dispersion behaviour between formulations containing 0.4 and 4% of drug is smaller with FLF added to the formulation with the lower drug content (Fig. 3A). This may be especially useful for formulations of the same brand and drug that are available in different dose strengths.

### About future perspectives

The carrier size fraction and the dispersion principle of the inhaler used may be important factors determining the balance between the different mechanisms in play, and therefore, there is a demand for studies focussing specifically on the interaction between these variables and the effects of added lactose fines on formulation dispersion performance. For example, the use of a finer carrier likely reduces the relevance of the press-on hypothesis and may also greatly affect the potential for agglomeration of the fine components on the carrier surface [19]. Furthermore, the test inhaler used in this study relies predominantly on inertial (collisional, vibrational and rotational) forces for the separation of drug particles from the carrier surface. This may cause agglomeration effects to be more dominant, and the difference between FLF and CLF to be more pronounced, than would be the case for dispersion principles relying on aerodynamic (drag, lift and shear type) forces. However, because beneficial and harmful effects of drug content on the fine particle fraction from different devices (dispersion principles) were observed previously [6], and because a great similarity between the effects of drug content and FLF was observed in this study, it is anticipated that the general findings from this study will apply to a range of different dispersion principles.

The fact that added lactose fines may either improve or deteriorate the dispersion performance of carrier-based inhalation powders means that a much broader perspective is needed than has previously been presented to obtain a mechanistic understanding of this formulation technique. Future investigations should be based on the idea that added fines may have any effect, which is determined by the formulation and dispersion conditions. This means that mechanistic studies in which just one fixed set of conditions is used can be considered obsolete from this moment onwards. Further unravelling of the significance of different possible mechanisms may also rely heavily on the development of new or improved techniques to measure relevant powder properties. Especially relevant properties may be the agglomeration behaviour of the fine components on the carrier surface, the tensile strength of fine particle networks and the spatial distribution of the different fine components over the carrier surface. In addition, the previously expressed desire for sensitive methods to measure mixture flowability remains [4].The influence of added fines on this mixture property was not considered in the current study, because its relevance to dispersion performance was minimised by directly weighing samples into the dispersion principle (i.e. the air classifier) of the test inhaler. However, for other devices (e.g. capsule based inhalers) the fluidisation hypothesis (Table 1) could well be relevant. Furthermore, fines may influence press-on effects during mixing through an influence on powder flowability.

## Conclusions

Added lactose fines are likely to exert their effect on the dispersion performance of carrier-based inhalation formulations through a combination of different mechanisms. The balance between the different mechanisms can be shifted by formulation and dispersion variables, such as the inhalation flow rate, the size distribution of the added fines, the drug content and the mixing order. Two new mechanisms may play a role in addition to those proposed previously. Firstly, it was shown that added lactose fines may lower dispersion performance, likely by increasing the effectiveness of press-on forces and the formation of coherent fine particle networks on the carrier surface. Secondly, a remarkable difference in effect on drug detachment between fine (X_50_ = 1.95 µm) and coarse lactose fines (X_50_ = 3.94 µm) may be explained by the coarse lactose fines weakening or preventing the formation of such coherent particle networks. It is of importance to the further mechanistic understanding of the effect of fines to better define ‘active sites’, to acknowledge that multiple mechanisms may play a role simultaneously and to study the interactions with other formulation and dispersion variables.

## References

[1] Arnold K, Grass P, Knecht A, Roos R, Sluke G, et al. (1995) Powders for inhalation. A61K 9/14 ed.

[2] Jones MD, Price R (2006) The influence of fine excipient particles on the performance of carrier-based dry powder inhalation formulations. Pharmaceutical Research 23: 1665–1674. 16845584 10.1007/s11095-006-9012-7

[3] de Boer AH, Chan HK, Price R (2012) A critical view on lactose-based drug formulation and device studies for dry powder inhalation: Which are relevant and what interactions to expect? Advanced Drug Delivery Reviews 64: 257–274. 21565232 10.1016/j.addr.2011.04.004

[4] Jones MD, Santo JGF, Yakub B, Dennison M, Master H, et al (2010) The relationship between drug concentration, mixing time, blending order and ternary dry powder inhalation performance. International Journal of Pharmaceutics 391: 137–147. 20211715 10.1016/j.ijpharm.2010.02.031

[5] Thalberg K, Berg E, Fransson M (2012) Modeling dispersion of dry powders for inhalation. The concepts of total fines, cohesive energy and interaction parameters. International Journal of Pharmaceutics 427: 224–233. 22349053 10.1016/j.ijpharm.2012.02.009

[6] Grasmeijer F, Hagedoorn P, Frijlink HW, De Boer AH (2013) Drug content effects on the dispersion performance of adhesive mixtures for inhalation. PLoS ONE 8: e71339. 23967195 10.1371/journal.pone.0071339 PMC3743805

[7] Lucas P, Anderson K, Staniforth J (1998) Protein Deposition from Dry Powder Inhalers: Fine Particle Multiplets as Performance Modifiers. Pharmaceutical Research 15: 562–569. 9587952 10.1023/a:1011977826711

[8] Louey MD, Stewart PJ (2002) Particle Interactions Involved in Aerosol Dispersion of Ternary Interactive Mixtures. Pharmaceutical Research 19: 1524–1531. 12425471 10.1023/a:1020464801786

[9] de Boer AH, Hagedoorn P, Gjaltema D, Goede J, Frijlink HW (2003) Air classifier technology (ACT) in dry powder inhalation: Part 1. Introduction of a novel force distribution concept (FDC) explaining the performance of a basic air classifier on adhesive mixtures. International Journal of Pharmaceutics 260: 187–200. 12842339 10.1016/s0378-5173(03)00250-3

[10] Islam N, Stewart P, Larson I, Hartley P (2004) Lactose Surface Modification by Decantation: Are Drug-Fine Lactose Ratios the Key to Better Dispersion of Salmeterol Xinafoate from Lactose-Interactive Mixtures? Pharmaceutical Research 21: 492–499. 15070101 10.1023/B:PHAM.0000019304.91412.18

[11] Dickhoff BHJ, de Boer AH, Lambregts D, Frijlink HW (2006) The effect of carrier surface treatment on drug particle detachment from crystalline carriers in adhesive mixtures for inhalation. International Journal of Pharmaceutics 327: 17–25. 16920287 10.1016/j.ijpharm.2006.07.017

[12] Kendall K, Stainton C (2001) Adhesion and aggregation of fine particles. Powder Technology 121: 223–229.

[13] Adi H, Larson I, Chiou H, Young P, Traini D, et al (2006) Agglomerate Strength and Dispersion of Salmeterol Xinafoate from Powder Mixtures for Inhalation. Pharmaceutical Research 23: 2556–2565. 16972185 10.1007/s11095-006-9082-6

[14] Donovan MJ, Kim SH, Raman V, Smyth HD (2012) Dry powder inhaler device influence on carrier particle performance. Journal of Pharmaceutical Sciences 101: 1097–1107. 22095397 10.1002/jps.22824

[15] Dickhoff BHJ, Ellison MJH, de Boer AH, Frijlink HW (2002) The effect of budesonide particle mass on drug particle detachment from carrier crystals in adhesive mixtures during inhalation. European Journal of Pharmaceutics and Biopharmaceutics 54: 245–248. 12191698 10.1016/s0939-6411(02)00082-6

[16] Zeng XM, Martin GP, Tee S-K, Ghoush AA, Marriott C (1999) Effects of particle size and adding sequence of fine lactose on the deposition of salbutamol sulphate from a dry powder formulation. International Journal of Pharmaceutics 182: 133–144. 10341303 10.1016/s0378-5173(99)00021-6

[17] Dickhoff BHJ (2006) Adhesive mixtures for powder inhalation. Groningen, The Netherlands: University of Groningen.

[18] Usmani OS, Biddiscombe MF, Barnes PJ (2005) Regional Lung Deposition and Bronchodilator Response as a Function of {beta}2-Agonist Particle Size. Am J Respir Crit Care Med 172: 1497–1504. 16192448 10.1164/rccm.200410-1414OC

[19] Grasmeijer F, Hagedoorn P, Frijlink HW, de Boer AH (2013) Mixing Time Effects on the Dispersion Performance of Adhesive Mixtures for Inhalation. PLoS ONE 8: e69263. 23844256 10.1371/journal.pone.0069263 PMC3699552

[20] Shur J, Harris H, Jones MD, Kaerger JS, Price R (2008) The role of fines in the modification of the fluidization and dispersion mechanism within dry powder inhaler formulations. Pharmaceutical Research 25: 1631–1640. 18239861 10.1007/s11095-008-9538-y

[21] Dickhoff BHJ, de Boer AH, Lambregts D, Frijlink HW (2003) The effect of carrier surface and bulk properties on drug particle detachment from crystalline lactose carrier particles during inhalation, as function of carrier payload and mixing time. European Journal of Pharmaceutics and Biopharmaceutics 56: 291–302. 12957644 10.1016/s0939-6411(03)00109-7

